# Preparation and characterization of novel as-cast Ti-Mo-Nb alloys for biomedical applications

**DOI:** 10.1038/s41598-022-14820-8

**Published:** 2022-07-13

**Authors:** Giovana Collombaro Cardoso, Gerson Santos de Almeida, Dante Oliver Guim Corrêa, Willian Fernando Zambuzzi, Marília Afonso Rabelo Buzalaf, Diego Rafael Nespeque Correa, Carlos Roberto Grandini

**Affiliations:** 1 Laboratório de Anelasticidade e Biomateriais, UNESP - Univ Estadual Paulista, Bauru, SP 17.033-360 Brazil; 2 IBTN-Br – Institute of Biomaterials, Tribocorrosion and Nanomedicine – Brazilian Branch,, Bauru, SP 17.033-360 Brazil; 3 Instituto de Biociências, UNESP - Univ Estadual Paulista, Botucatu, SP 18.618-689 Brazil; 4 Bauru School of Dentistry, University of São Paulo, Bauru, SP 17.012-901 Brazil; 5 IFSP – Federal Institute of Education, Science and Technology of São Paulo, Sorocaba, SP 18.095-410 Brazil

**Keywords:** Engineering, Materials science, Physics

## Abstract

Ti and its alloys are the most used metallic biomaterials devices due to their excellent combination of chemical and mechanical properties, biocompatibility, and non-toxicity to the human body. However, the current alloys available still have several issues, such as cytotoxicity of Al and V and high elastic modulus values, compared to human bone. β-type alloys, compared to α-type and (α + β)-type Ti alloys, have lower elastic modulus and higher mechanical strength. Then, new biomedical β-type alloys are being developed with non-cytotoxic alloying elements, such as Mo and Nb. Therefore, Ti-5Mo-xNb system alloys were prepared by argon arc melting. Chemical composition was evaluated by EDS analysis, and the density measurements were performed by Archimedes' method. The structure and microstructure of the alloys were obtained by X-ray diffraction and optical and scanning electron microscopy. Microhardness values were analyzed, and MTT and crystal violet tests were performed to assess their cytotoxicity. As the Nb concentration increases, the presence of the β-Ti phase also grows, with the Ti-5Mo-30Nb alloy presenting a single β-Ti phase. In contrast, the microhardness of the alloys decreases with the addition of Nb, except the Ti-5Mo-10Nb alloy, which has its microhardness increased probably due to the ω phase precipitation. Biological in-vitro tests showed that the alloys are not cytotoxic.

## Introduction

Nowadays, the number of older people is increasing worldwide. Consequently, the demand for materials to replace hard tissues, such as hip and knee implants, also increases to provide a better life quality and clinical treatment for age-related diseases^1,2^. Due to their excellent properties, such as high mechanical strength, good resistance to corrosion, low elastic modulus, and excellent biocompatibility, Ti alloys are widely used in biomedical applications^3^. CP-Ti and Ti-6Al-4 V alloy are broadly used as implant materials^4^. However, studies have shown that V ions are cytotoxic and can cause adverse reactions in the body, while Al ions can induce neurological disorders, such as Alzheimer's disease^2,5^. Therefore, new alloys without Al and V are being developed, maintaining the already known properties of Ti alloys. To overcome this issue, non-toxic and non-allergenic β-stabilizing elements, such as Ta, Zn, Sn, Nb, and Mo, are used. These elements produce Ti alloys with high mechanical strength and low elastic modulus^6^.

Although some studies indicate that the Mo ion releasing can be toxic^7,8^, others show that Ti alloys containing Mo present excellent biocompatibility^9^, such as Ti-15Mo^10^, Ti-15Mo-5Mn^11^, and Ti-12Mo-6Zr-2Fe (TMZF)^12^. Other studies have shown that Ti alloys containing Mo exhibit good mechanical compatibility^13^, such as Ti-Mo^14^ and Ti-Mo-Ta^15^. Also, Karthega et al.^16^, Oliveira et al.^17^, and Zhou et al.^18^ showed that Ti-Mo alloys have excellent corrosion resistance against simulated body fluids^13^. Other studies have shown that Ti-Mo-Nb alloys presented good mechanical properties, corrosion resistance^19–21^, and adequate *in-vitro* biocompatibility^22^. Mo is also a strongly β-stabilizing element. Therefore, high concentrations can lead to increased atomic binding energy, tending to increase the modulus of elasticity of the alloys^23^. Thus, due to the low number of studies with ternary alloys of Ti-Mo-Nb with low Mo concentration, varying the Nb from low to high contents, and aiming to avoid an increase in the modulus of elasticity of the alloys and possible cytotoxicity of Mo, it was chosen to work with only 5% by weight of Mo element in the alloys.

In addition, Brazil has about 90% of the world's niobium resources, accounting for about 95% of worldwide production. In this way, from an economic and strategic perspective, it is crucial to invest in research relating to the processing and development of alloys containing niobium, as Brazil leads the world's resources of this metal^24,25^.

In this study, a novel Ti-Mo-xNb (x = 0, 10, 20, and 30 wt%) alloys system was prepared by argon arc melting to evaluate the effect of Nb on the mechanical properties of alloys with a low amount of Mo.

## Results and discussion

**Figure 1:**
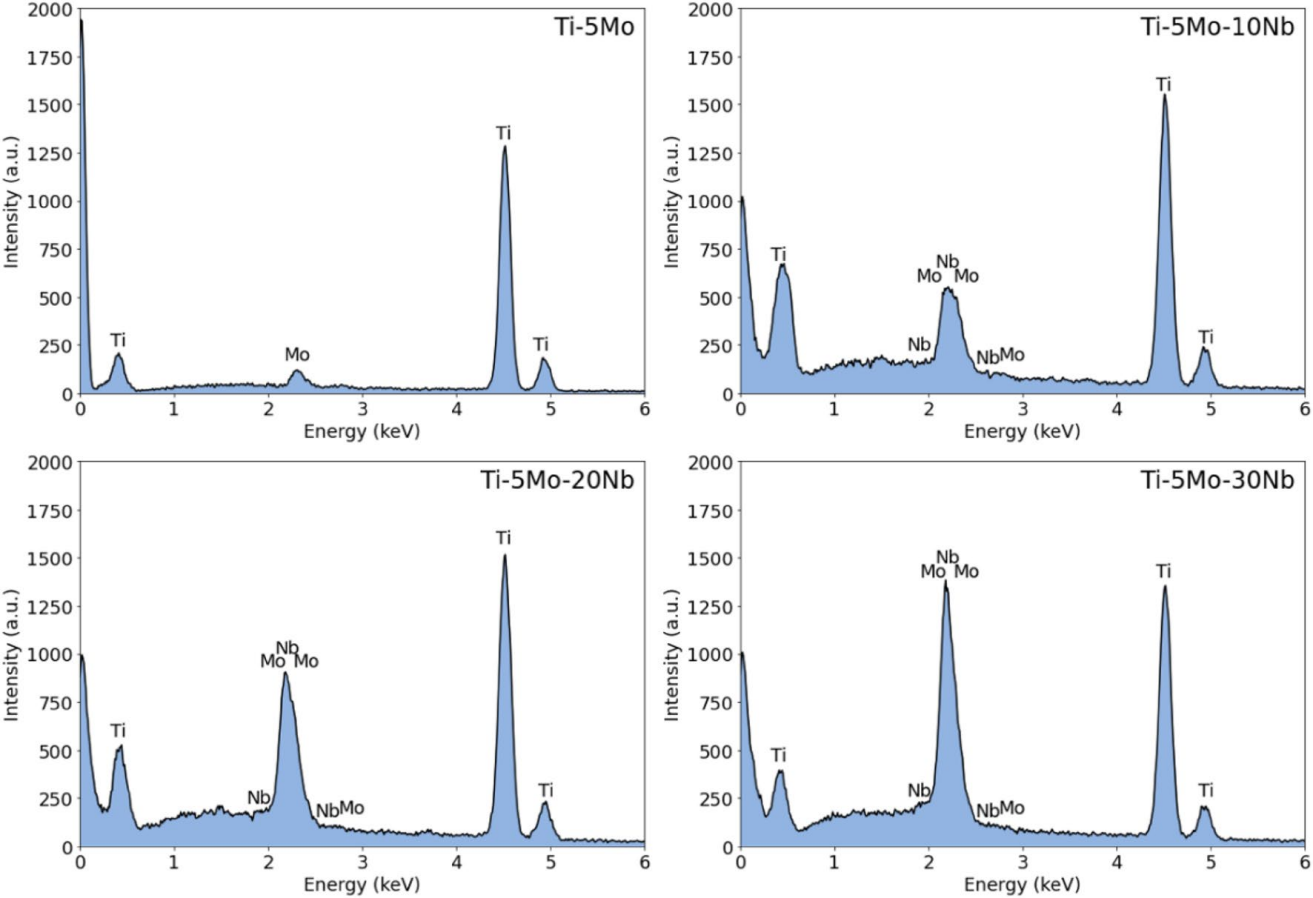
Qualitative chemical analysis of as-cast Ti-5Mo-Nb system alloys by EDS.

**Figure 2:**
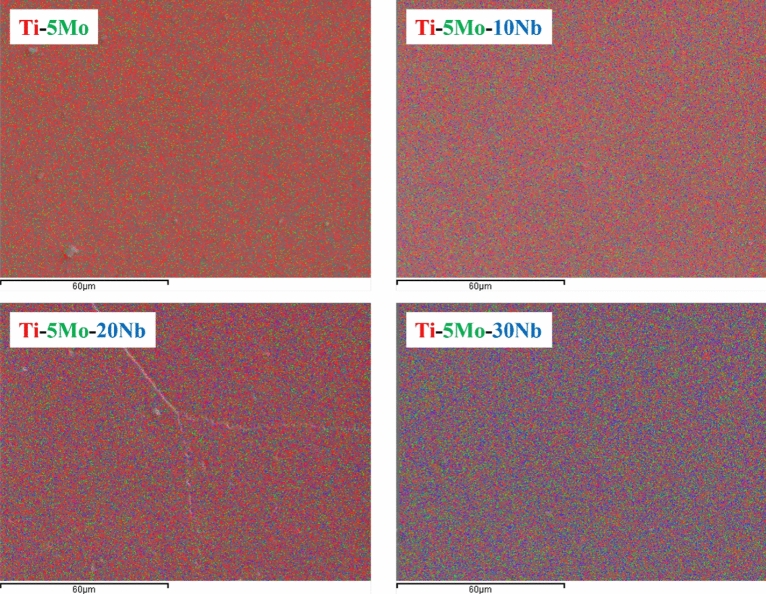
Chemical mapping of the elements Ti (red), Mo (green), and Nb (blue) of the as-cast Ti-5Mo-Nb system alloys by EDS.

Table 1 presents the chemical composition obtained by EDS. It is possible to observe that the chemical composition remained close to the nominal values. Figure 1 shows the EDS spectrum for each studied as-cast ingot, where only peaks of the alloy elements (Ti, Mo, and Nb) are observed, indicating a good quality of the produced samples. Figure 2 shows the EDS elemental mappings of each produced alloys after melting. It is observed that the elements are well distributed. No agglomerated and segregated elements were observed, showing the excellent homogeneity of the ingots. For better visualization, the elements of the studied system were labeled with different colors, Ti as red, Mo green, and Nb blue.

**Table 1:** Semiquantitative chemical composition of the Ti-5Mo-Nb system alloys, by EDS.

Alloy	Ti (wt%)	Mo (wt%)	Nb (wt%)
Ti-5Mo	93.8 ± 0.4	6.2 ± 0.4	-
Ti-5Mo-10Nb	84.0 ± 0.9	5.3 ± 0.5	11.0 ± 0.6
Ti-5Mo-20Nb	73.7 ± 0.9	5.2 ± 0.9	21.3 ± 0.3
Ti-5Mo-30Nb	63.0 ± 0.5	5.8 ± 0.7	31.2 ± 0.4

**Figure 3:**
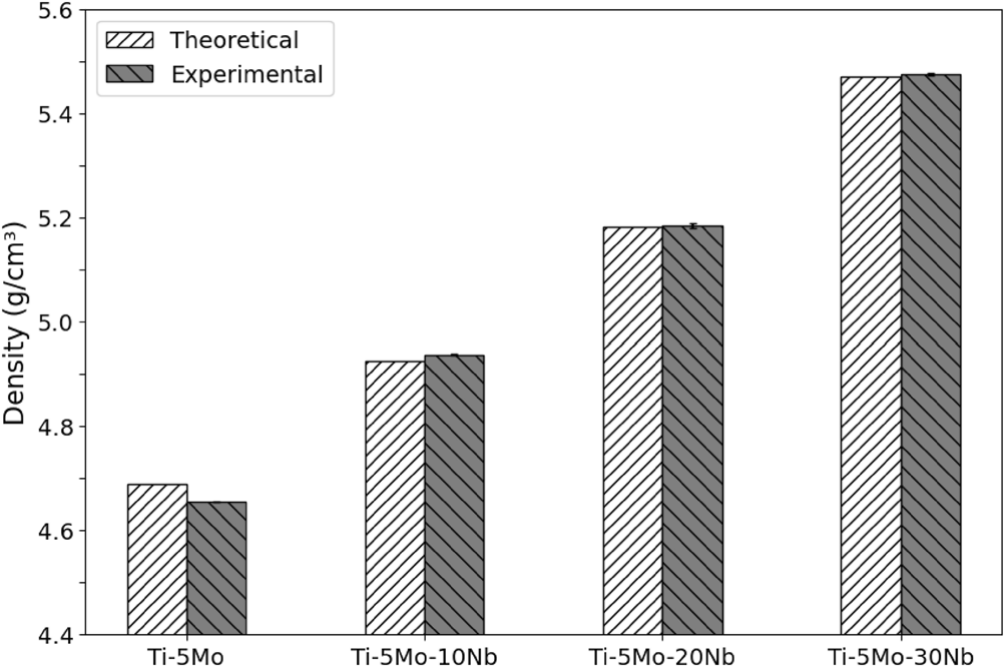
Comparison between the theoretical and experimental densities of the Ti-5Mo-Nb system alloys.

Figure 3 shows the comparison between the measured density and the alloys' respective calculated theoretical density. The theoretical and experimental density remained close, evidence of a suitable stoichiometry of the produced alloys.

An increase in density is observed as the Nb content of the alloys increases, ranging from 4.66 g/cm^3^, for Ti-5Mo alloy, to 5.48 g/cm^3^ for Ti-5Mo-30Nb alloy. This increase was due to the density of the alloying elements Mo (10.22 g/cm^3^) and Nb (8.58 g/cm^3^) being higher than the density of Ti (4.54 g/cm^3^)^26^.

The density values of the studied alloys remained low if compared with other metallic biomaterials, such as AISI 316L (7.93 g/cm^3^) and CoCr alloys (9.2 g/cm^3^), and the values are relatively close to the CP-Ti (4.5 g/cm^3^) and Ti-6Al-4 V alloy (4.42 g/cm^3^)^27^.

**Figure 4:**
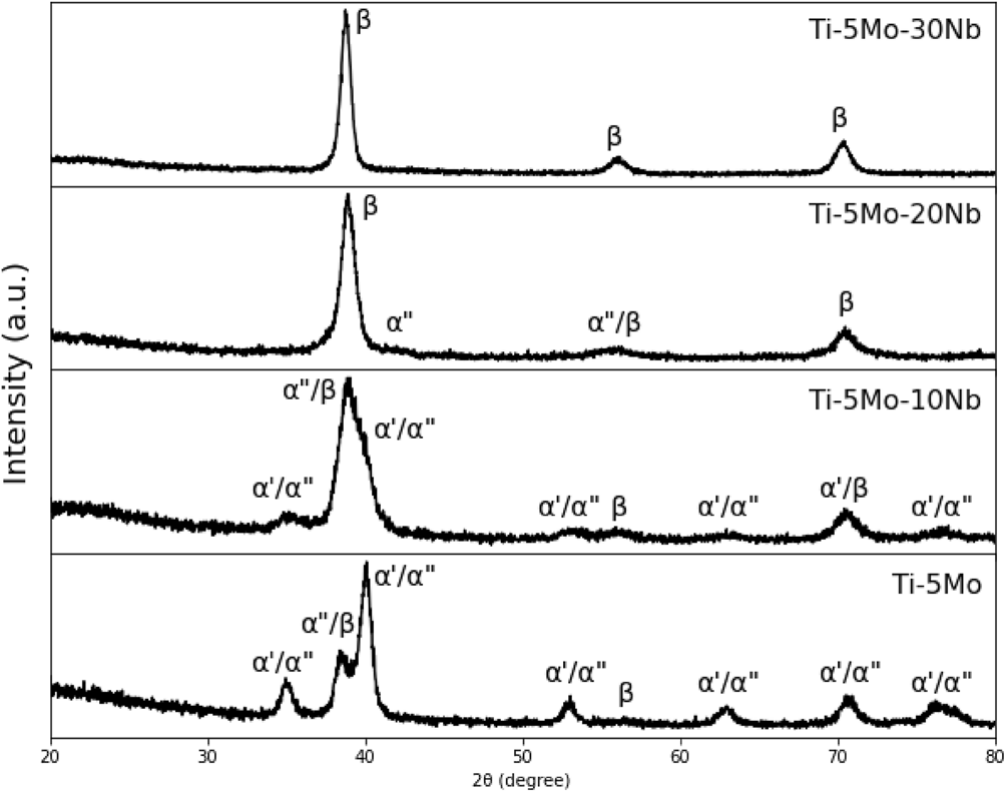
X-ray diffractograms of as-cast Ti-5Mo-Nb system alloys.

The X-ray patterns are shown in Fig. 4. The as-cast Ti-5Mo alloy is mainly composed of the α' phase (63%), also presenting 22% of the α" phase and a small amount of β phase (15%). When adding 10% by weight of Nb to the alloy, there is still the presence of the three phases of Ti, but the amount of α" and β phases increased to 50% and 38%, respectively, with a decrease to 12% of the α' phase. The Ti-5Mo-20Nb alloy predicted as a metastable β alloy showed peaks of the α" orthorhombic phase (40%) and the β phase (60%). Finally, the Ti-5Mo-30Nb alloy only presented peaks of the β phase. Thus, it was observed that, with the Nb addition, there is an increase in the β phase content in the microstructure of the Ti-5Mo-xNb system alloys, and with 30% by weight of Nb, it is possible to obtain an alloy with a predominance of β phase after melting.

Although simpler representations of the Ti-Mo^28,29^ and Ti-Nb^30,31^ alloys exist, the diagram proposed by Zhang et al^30,31^ presents the wide range of transient states and phase transformations due to the wide α + β field. For low temperatures, ω phase formation is shown for all Nb concentrations. α' and α" martensite phase formation were further added to the diagram, respectively, for concentrations below and above 14% by weight of Nb^30^. This phase change from α' to α" happened with the studied alloys between 10 and 20% by weight of Nb.

High cooling rates form the α' martensite phase from the β-phase field. Its microstructure, characterized by a coarse acicular morphology, comes from the lack of diffusional growth from the β to α phase^32^. The martensitic α" phase, also characterized by an acicular morphology but thinner than the α' phase, can be generated by the application of external mechanical deformation or high cooling rates and is formed when the concentration of β-stabilizing elements is higher than in the α' phase^32^. The ω phase can be formed by mechanical deformation, rapid cooling, or thermal aging treatment^32,33^.

**Figure 5:**
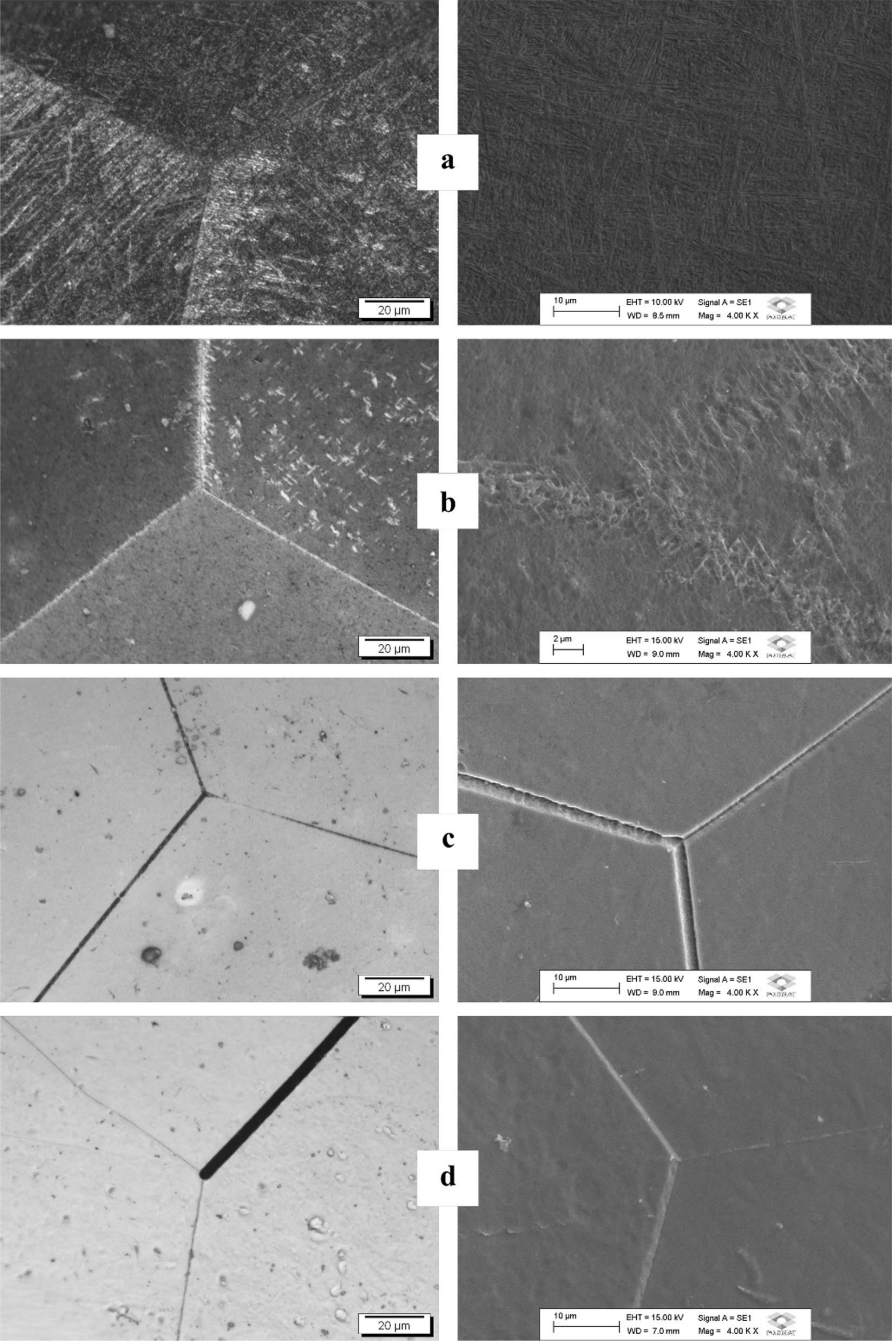
OM, with 1000 × magnification (left), and SEM, with 4000 × magnification (right) of as-cast Ti-5Mo (a), Ti-5Mo-10Nb (b), Ti-5Mo-20Nb (c) alloys and Ti-5Mo-30Nb (d) alloys.

The OM and SEM images are presented in Fig. 5 and corroborate with the X-ray results. In the microstructure of the Ti-5Mo and Ti-5Mo-10Nb alloys, fine and coarse acicular needles were identified, typical of the α" and α' phases, respectively, in addition to grain boundaries characteristic of the β phase. The Ti-5Mo-20Nb and Ti-5Mo-30Nb alloys have only equiaxed grain boundaries characteristic of the β phase^34,35^.

**Figure 6:**
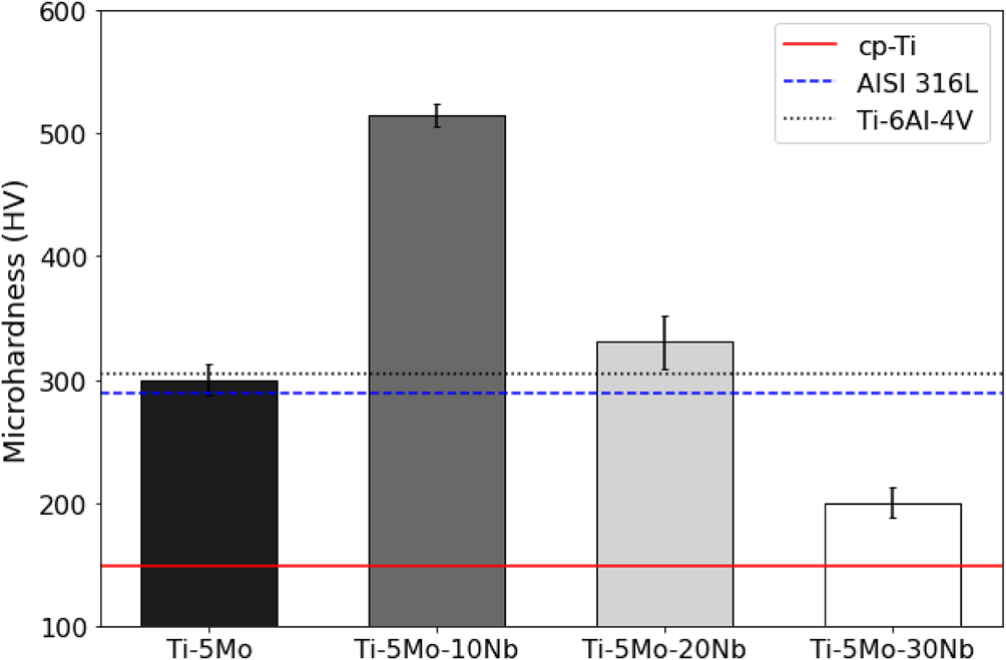
Vickers Microhardness of as-cast Ti-5Mo-xNb system alloys, compared to other metallic biomaterials.

The Vickers microhardness values of the alloys are shown in Fig. 6. All the alloys have a microhardness greater than the CP-Ti (148 HV), as shown in the red reference line. With the addition of 10% Nb, the microhardness value increases from 300 to 515 HV. Xu et al*.*^19^ studied the Ti-15Mo-xNb system alloys and also obtained an increase in the microhardness value of the alloy with 10% of Nb, which can be caused by the formation of ω phase, which makes the material harder and brittle^36–38^. With the addition of 20% and 30% Nb, there is a reduction in the microhardness values to 330 HV and 200 HV, respectively. This reduction can be explained by the increase of the β phase in these alloys, which tends to decrease the hardness values of the alloys since the increase of β-stabilizer elements reduces the chemical bond strength, facilitating the plastic deformation^39–41^. The microhardness reduction of the alloys is important because it facilitates their mechanical conformation^39^. The microhardness of Ti-5Mo alloy remained close to the AISI 316L (289 HV) and Ti-6Al-4 V (304 HV), while the Ti-5Mo-30Nb alloy remained well below these same metallic biomaterials.

The structure of bones is dynamic, and their tissues can vary depending on the load applied^42^. Table 2 presents the Vickers microhardness values of the alloys studied compared to the average of human cortical bone.

**Table 2:** Vickers microhardness of as-cast Ti-5Mo-XNb and cortical bone.

Material	Average Vickers Microhardness (HV)
Ti-5Mo (as-cast)	300 ± 13
Ti-5Mo-10Nb (as-cast)	515 ± 9
Ti-5Mo-20Nb (as-cast)	330 ± 22
Ti-5Mo-30Nb (as-cast)	200 ± 13
Cortical bone^43^	40

**Figure 7:**
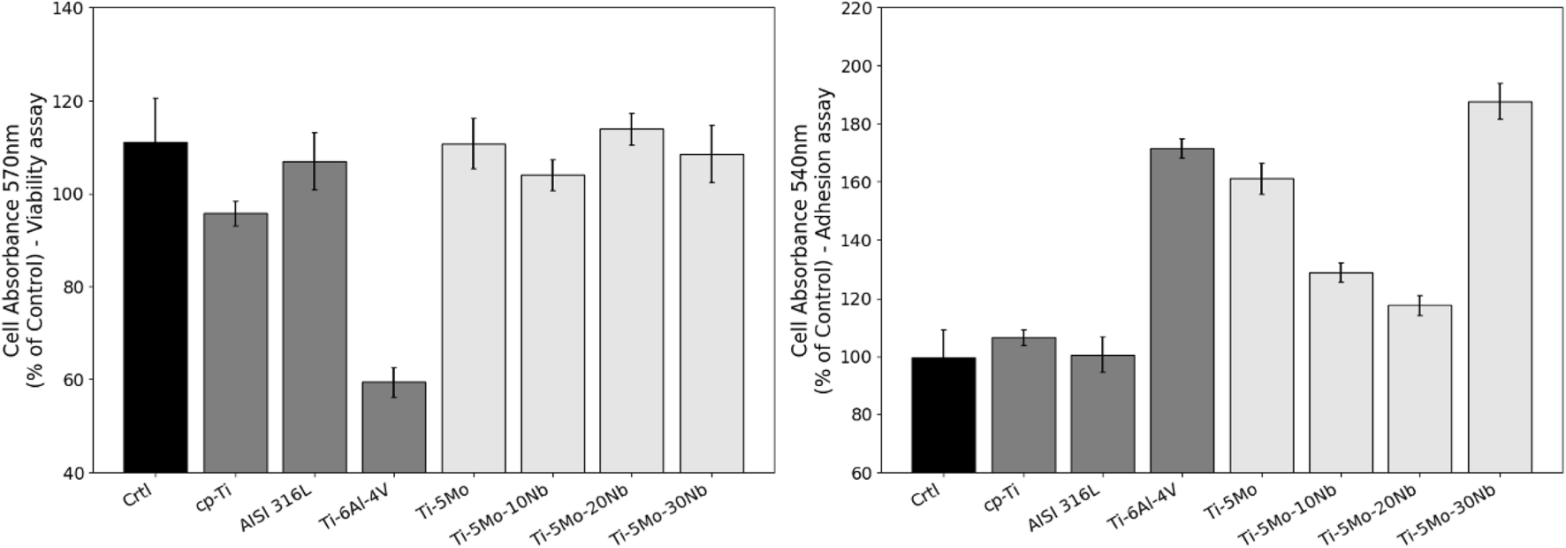
Cell viability (left) and adhesion (right) assays of as-cast Ti-5Mo-Nb system alloys and some other metallic biomaterials.

Using the ISO10993 standard^44^, a biomaterial will only be considered cytotoxic if cell viability is below 70%. The studied alloys did not show a cytotoxicity effect (Fig. 7, left panel). The cells in the culture medium remained viable even after being conditioned to the alloys, which can be observed by activating the cellular mitochondrial pathway, which MTT analyzes.

Regarding cell adhesion, all analyzed alloys maintained percentages above the control group, as presented in Fig. 7, showing a stimulus for cell adhesion in the analyzed culture medium, an essential factor for cell survival. The differences found when the alloys studied were compared could be related with the capacity of them in interacting with the cell culture medium once they are able to release elements with further dynamic capacity in modifying cell performance, as we have reported earlier by evaluating other alloys^45–47^. Briefly, released elements from the alloys must drive different intracellular signaling mainly those related upon integrin activation and requiring the balance of actions of kinases and phosphatases^48,49^, but new experimental approaches need to be considered further to better comprehend this mechanism. However, it is necessary to mention that all those alloys investigated promoted higher adhesion when they were compared with the control, when the cells were maintained under classical cell culture conditions.

## Conclusions

With the obtained results, it follows that:

- Semi-quantitative chemical composition by EDS confirms the excellent quality of the ingots produced and that the constituent elements of the samples are close to the nominal compositions initially proposed.
- The samples' chemical mapping shows no agglomerates or segregated elements, indicating good homogeneity of the ingots produced.
- The density of the alloys remained low and close to the CP-Ti, increasing as the amount of niobium increased.
- The microstructure of the alloys proved to be sensitive to the addition of Nb: the amount of β phase increased as the Nb content of the alloys increased, having the Ti-5Mo-30Nb alloy presented only this phase.
- The Vickers microhardness values decreased with the increase of Nb, except the Ti-5Mo-10Nb, which had its microhardness increased, probably due to the presence of ω phase in its microstructure.
- Cytotoxicity tests show that the alloys have no cytotoxic effect and keep the cells viable, causing the stimulation for cell adhesion, which indicates that the alloys have great potential as biomaterials to be used in the health area. Contact angle measurements can provide a better analysis of cell adhesion.

## Materials and methods

The samples were produced by using commercially pure Ti grade 2 (CP-Ti, Sandinox), Mo (99,9% purity, Sigma-Aldrich), and Nb (99,8% purity, Sigma-Aldrich) as precursor materials. The metals were separated in the nominal composition of each alloy. Ingots with approximately 60 g were melted using an arc-melting furnace with a tungsten electrode in an inert and controlled argon gas atmosphere. To ensure the homogeneity of each sample, the ingots were remelted five times.

After melting, the ingots were chemically characterized, with chemical composition measurements performed by the Energy Dispersive Spectroscopy (EDS) method, using an Oxford, INCA model detector coupled in SEM equipment. In addition, the densities values of the samples were measured by Archimedes' method in a digital balance.

Structural and microstructural analyses were performed by X-ray diffractometry (XRD), optical microscopy (OM), and scanning electron microscopy (SEM). The XRD measurements were performed on a Panalytical X'Pert-Pro model, with Cu-Kα radiation, 30 mA current, 40 kV potential, and continuous-time mode. OM and SEM images were obtained in an optical microscope (Olympus BX51M model) and a Carl Zeiss microscope (EVO-015 model). Rietveld refinement of the XRD patterns was performed by the GSAS software^50^, with the EXPEGUI interface^51^, using the crystallographic datasheets of metallic titanium phases^52^, and a standard Ti–cp sample was used to eliminate the experimental contribution of the equipment^23^.

Microhardness measurements were obtained in Shimadzu HMV-2 model equipment, with five indentations in each sample, a load of 25 gf, and 10 s of duration. The measurements were made based on the ASTM E92 standard^53^.

Biological tests of MTT and crystal violet were performed to verify cell viability and adhesion when in contact with samples of the produced alloys. To verify the cytotoxic potential of the alloys, they were kept in cell culture for 24 h, following the ISO 10,993 standard^44^. After the conditioning period, the cell culture medium (αMEM; Sigma) was collected and supplemented with 10% Fetal Bovine Serum (FBS) (Nutricell, Campinas, SP, Brazil) and used to treat pre-osteoblasts for 24 h. The pre-osteoblasts (MC3T3-E1, subclone 4) were obtained from ATCC and maintained in this study as recommended by the manufacturer guidelines. Briefly, the cells were maintained at 37ºC and 95% humidity in 5% CO2 in an incubator with a specific cell culture medium containing antibiotics (100 U/mL penicillin, 100 mg/mL streptomycin) supplemented with 10% Fetal Serum Bovine. Cells were plated 24 h before treatment in a 96-well plate and at a 5 × 10^4^ cells/ml density. After the determined time of exposure to the conditioned cell culture, the viability of these cells was measured using the MTT test. Where the culture medium was removed, 1 mg/ml Thiazolyl Blue Tetrazolium Bromide salt (Sigma Aldrich #M5455-1G) was added and placed in an oven for an additional 3 h. After this period, the cell culture was removed, and 0.1 ml of DMSO was added to solubilize the dye formed by the viable cells. Afterward, the absorbance was measured at 570 nm using a Biotek SYNERGY-HTX multi-mode microplate reader.

For cell adhesion assays, pre-osteoblasts were plated with media conditioned by the alloys. Cells were seeded into 96-well plates at a density of 5 × 10^4^ cells/ml. After 24 h, the medium was removed, and adhesion was measured by incorporating Crystal Violet. Absorbance was measured at 540 nm in a Biotek microplate reader. Results were represented as mean ± standard deviation (SD). They were verified using One-Way ANOVA (parametric) with Tukey post-test to compare all pairs of groups. In this case, p < 0.05 was considered statistically significant, and *p* < 0.0001 was considered highly significant. The software used was GraphPad Prism 7.

## Data Availability

The data that support the findings of this study are available from the corresponding author.
